# Development of a two-tube multiplex real-time fluorescent PCR for the simultaneous differentiation of the mpox virus clades and the A.1, B.1 and C.1 lineages within clade IIb

**DOI:** 10.3389/fcimb.2025.1611248

**Published:** 2025-10-01

**Authors:** Guohao Fan, Yuanlong Lin, Liuqing Yang, Yun Peng, Guanyong Ou, Qi Qian, Dongmei Lai, Fuxiang Wang, Yingxia Liu, Yang Yang

**Affiliations:** ^1^ Shenzhen Key Laboratory of Pathogen and Immunity, Shenzhen Clinical Research Center for Infectious Disease, State Key Discipline of Infectious Disease, Shenzhen Third People’s Hospital, Second Hospital Affiliated to Southern University of Science and Technology, Shenzhen, China; ^2^ National Clinical Research Center for Infectious Disease, Shenzhen, China; ^3^ Guangdong Key Laboratory for Diagnosis and Treatment of Emerging Infectious Diseases, Shenzhen, China

**Keywords:** Mpox virus, multiplex qPCR, clade Ib, locked nucleic acid (LNA), diagnosis

## Abstract

**Background:**

New clades and lineages emerged with the globally prevalent of Mpox virus (MPXV), accompanied by changing clinical symptoms, pathogenesis and transmission dynamics in associated with specific clades and lineages.

**Methods:**

Here, we developed a two tube multiplex real-time fluorescent quantitative PCR (mrt-qPCR) assay for simultaneous differentiation of MPXV clades Ia, Ib, II, and innovative binding lock nucleic acid (LNA) probes to detect A.1, B.1 and C.1 lineages within the clade IIb.

**Results:**

The assay demonstrated high sensitivity (33–69 copies/reaction) and specificity with expected linearity and stability. The intra-assay and intre-assay coefficients of variations (CV) were below the acceptable threshold of 5%, and the mrt-qPCR method has good stability and reproducibility. Clinical validation using 109 qPCR positive, 1 clade IIb B.1 virus strain and 15 negative specimens revealed 100% concordance for the differentiation of the three clade II and 97.60% for the differentiation the three lineages. The two tube multi-test system streamlined workflows, enabling efficient screening of diverse clinical samples (swabs from skin lessions, oropharynx and rectum, saliva and plasma).

**Conclusions:**

We have established a two-tube multiplex qPCR method for detecting different clades and lineages of the MPXV. This method addresses the issue of false-negative detection of MPXV clade Ib caused by gene fragment deletion, and has also enabled the development of a rapid detection approach for the predominantly circulating clade IIb (including lineages A.1, B.1, and C.1). This cost-effective assay provides an important tool for accurate diagnosis, typing and epidemiological surveillance of MPXV.

## Introduction

Mpox is a zoonotic disease caused by Mpox virus (MPXV), a large double-stranded DNA virus belonging to the genus Orthopoxvirus (Fan et al., 2024; Moss, 2024). MPXV was isolated and identified from smallpox-like skin lesions in captive cynomolgus monkeys in 1958 (Magnus et al., 1959). Human infections were first reported in Central and West Africa in the 1970s (Moss, 2024), then human cases have been documented predominantly in Central and West African countries, which are regarded as endemic regions for MPXV (Thornhill et al., 2022). As the globally prevalent MPXV emerged, it gave rise to new clades and lineages. This emergence was accompanied by alterations in clinical symptoms, pathogenesis and transmission dynamics, in association with specific clades and lineages (Duarte et al., 2024). A notable outbreak of MPXV that began in 2022 has spread at an accelerated rate across the globe, affecting over 130 countries that had not previously reported cases of Mpox (Angelo et al., 2023; Fan et al., 2024). This outbreak has demonstrated a disproportionate impact on communities of men who have sex with men (MSM) (Moss, 2024). The strain of MPXV that circulated during the 2022 outbreak is attributed to the B.1 lineage of the clade IIb (Song et al., 2025). During the course of the clade IIb epidemic, MPXV continued to evolve new lineages, with the early epidemic being primarily A.1, and then accumulating a number of lineage mutations based on A.1, evolving the B.1 lineage, which is the predominant endemic strain in 2022. During 2023, MPXV continued to spread and evolve, accumulating more columns of mutations before evolving the C.1 lineage, which is now prevalent and spreading, mainly in Portugal and China (Zhang et al., 2024). Consequently, accurate detection of the specific MPXV lineage is of great significance for the prevention of epidemics and for tracing the end of an epidemic.

A novel strain of MPXV, classified as a highly differentiated subtype of clade Ib, has emerged in an outbreak that commenced in September 2023 in the South Kivu province of the Democratic Republic of Congo (DRC) (Multi-country outbreak of mpox, External situation report, 2025). To date, imported cases of clade Ib have been reported in 12 countries outside Africa, including Sweden, Thailand, India, the United Kingdom, Germany, the United States, Canada, Belgium, France, Pakistan, Oman and China [China Center for Disease Control and Prevention (CDC)]. The genomic analysis of the newly detected clade Ib genome revealed a deletion of the target sequence (within the C3L gene) currently used for identifying clade I viruses (Schuele et al., 2024). This deletion would affect the accuracy of real-time qPCR detecting MPXV, leading to false negative identification of novel clade Ib lineage (Masirika et al., 2024).

MPXV is transmitted by a variety of routes and infected individuals may present with symptoms at different sites on the body, requiring diagnostic tools capable of detecting the virus from a variety of clinical specimens (Li et al., 2010; Huo et al., 2022; Low et al., 2023). In addition, genomic variation in MPXV can render certain diagnostic targets ineffective, so the development of multiplexed assays that can cover multiple targets is needed to improve diagnostic accuracy and robustness. Real-time quantitative fluorescent PCR (qPCR) is the most widely used and powerful tool for the diagnosis of MPXV because of its high sensitivity, specificity, low cost and well-established platform (Huggett et al., 2022; Schuele et al., 2024).

Therefore, we developed two tube multiplex real-time fluorescence quantitative polymerase chain reaction (mrt-qPCR) assays for the simultaneous detection and differentiation of MPXV clade Ia, Ib and II, and lock nucleic acid (LNA) probes for detection of clade IIb A.1, B.1 and C.1 lineages.

## Methods

### Sample collected and nucleic acid extraction

A total of 124 clinical specimens were collected from the Department of Infectious Diseases, Shenzhen Third People’s Hospital, Shenzhen, China, and specimen types included swabs from skin lessions, oropharynx and rectum, saliva and plasma. The nucleic acid were extracted using TIANamp Genomic DNA Kit (Tiangen Biotech Beijing Co., ltd.) according to the manufacturer’s instructions and stored in a refrigerator at -80 °C until use (Supplementary information).

### Preparation of recombinant plasmids

The target genes of clade Ia, Ib II, A.1, B.1 and C.1 were respectively cloned into vector pUC57 for recombinant plasmids (Shanghai Sangong Biological Engineering Co). The plasmid DNA was quantified using a Qubit 2.0 fluorometer (Life Technologies, Warrington, UK). The resulting DNA concentrations were converted to genome copy values using the formula: N (copy number/μL) = [6.02 × 10^23^ × n × 10^-9^]/[(target genes length + pUC57 length) × 660]. The plasmids were 10-fold diluted from 10^8^ to 10^1^ copies/μL, and stored at -20 °C.

N:DNA copy number. n: plasmid concentration (ng/μL).

### Primers and probes

**Figure 1:**
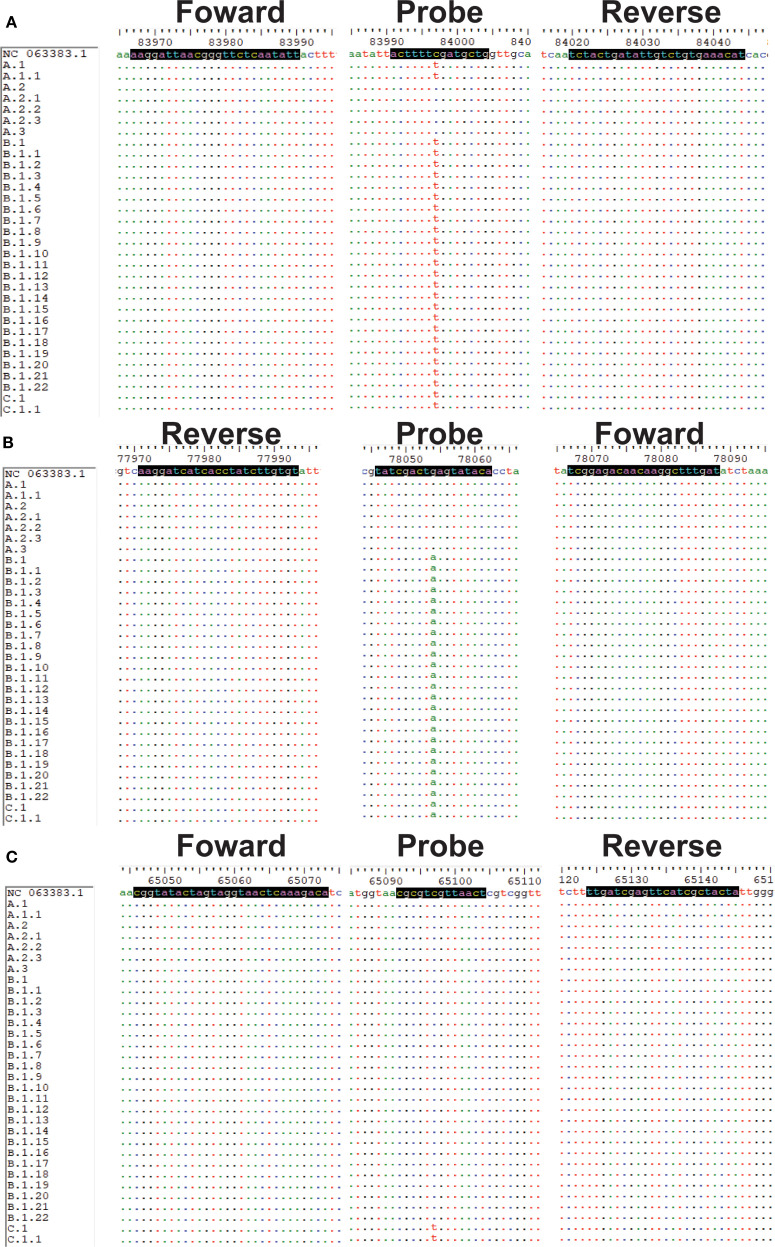
Sequence alignments of target regions for the MPXV (A.1, B.1 and C,1). **(A-C)** Sequences and positions of primers and probes for A.1, B.1 and C.1 lineages, respectively.

Sequences and specific primers and probes for MPXV (clade Ia, Ib, II, and clade IIb A.1, B.1 and C.1 lineages) were obtained using MAFFT 7.520 and Oligo 7, respectively. In particular, in Panel 1, the design of clade Ia and clade Ib primers and probes was such that they were situated within the deletion region of the genome. Moreover, the probes specific to clade Ib were designed to be positioned upstream and downstream of the aforementioned deletion region (**Supplementary Figure S1**). In Panel 2, the design of primers and probes was informed by the specific mutation sites of clade IIb A.1, B.1, and C.1 (**Figure 1**) (lineage-designation/auto-generated/lineages.md at master · mpxv-lineages/lineage-designation). Additionally, LNA modification was performed on the probes. The implementation of LNA modification on the bases has been demonstrated to enhance the specificity of the assay and elevate the Tm value of the probes (Chowdhury S. et al., 2022). This in turn shortens the length of the probes, allowing more precise detection of the mutation sites. The forward and reverse primers were designed to amplify these regions (**Figure 1**; **Supplementary Figure S1**). All primers and probes were synthesized by Shanghai Sangong Biological Engineering Co (**Supplementary Table S1**).

### Multiplex real-time fluorescent quantitative PCR assays

**Figure 2:**
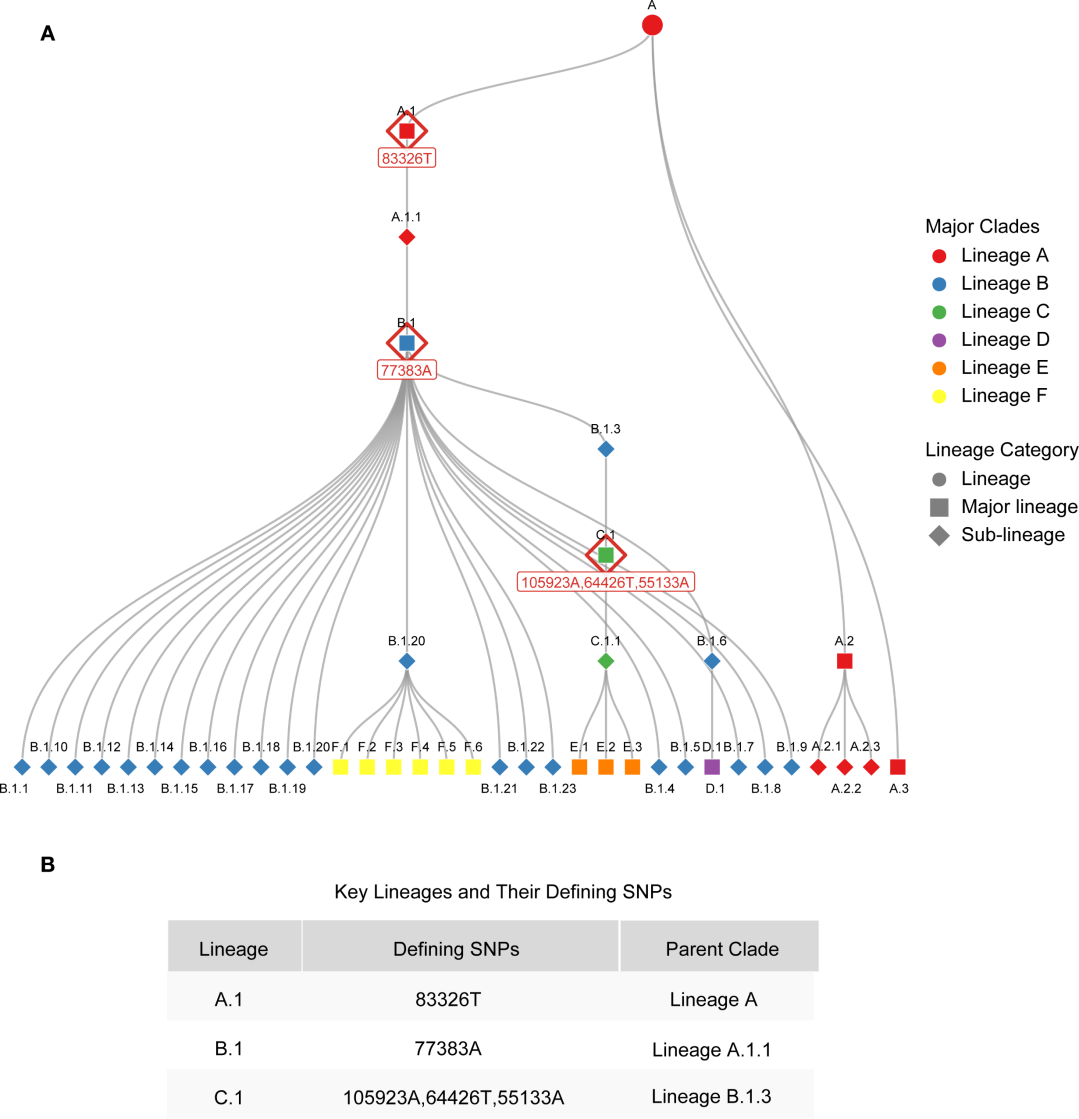
Phylogenetic relationships of MPXV lineages and key genetic markers of MPXV lineages. **(A)** Phylogenetic relationships of MPXV lineages. **(B)** Key genetic markers of A.1, B.1 and C.1 lineages.

In panel 1 (clade Ia, clade Ib and clade II), the mrt-qPCR assay was performed in 20 μL reaction volumes, contained 10 μL of 2 × Taq Pro U+ Probe qPCR Mix (Vazyme‐innovation in enzyme technology), 0.7 μL of each forward primers (10 μM), 0.7 μL of each reverse primers (10μM), 0.7 μL of each probers, 2 μL of template DNA (plasmids:1×10^8^-1×10^1^ copie/μL or nucleic acid of clinical sample), and 1.7 μL of RNase‐free and DNase‐free water. In panel 2 (lineage A,1, lineage B.1 and lineage C.1), the mix is analogous to panel 1, with the exception that the primers and probes are substituted with those of the targets detected by panel 2. The mrt-qPCR assay was performed using a LightCycler 96. The following thermal cycling program was used: 95°C for 5 min, followed by 45 cycles at 95°C for 10 s, 58°C for 30 s and 60°C for 30 s. The performance of mrt-qPCR was evaluated using a standard curve. It is worth noting that since clade IIb C.1 evolved from B.1, and B.1 evolved from A.1, the mutation site in A.1 is inherited in B.1 and C.1, and the mutation site in B.1 is inherited in C.1 (lineage-designation/auto-generated/lineages.md at master · mpxv-lineages/lineage-designation) (**Figure 2**; **Supplementary Table S2**). Therefore, the judgment criteria of Panel 2 are as follows: when lineage A,1, lineage B.1 and lineage C.1 are positive, the result is C.1 lineage; when lineage A,1, lineage B.1 is positive and lineage C.1 is negative, the result is B.1 lineage, and when lineage A,1 is positive, lineage B.1 and lineage C.1 are negative, the result is A.1 lineage.

### Analytical sensitivity, specificity, and reproducibility of the mrt-qPCR

Recombinant plasmids containing target sequences of MPXV clade Ia, clade Ib, and clade II (Panel 1), as well as clade IIb A.1, B.1, and C.1 (Panel 2), were employed as templates to assess sensitivity and reproducibility. Ten-fold serial dilutions were prepared in TE buffer (10 mM Tris-HCl, 1 mM EDTA, pH 8.0), covering a concentration range from 1.0 × 10⁸ to 1.0 × 10¹ copies/μL. Each dilution was assayed in 20 replicates (n = 20). Probit regression analysis was then performed to calculate their sensitivity.

Specificity was validated using the following approaches: ectromelia virus (ECTV) was used to evaluate assay specificity for monkeypox virus detection, while clinical samples of MPXV clade IIb C.1 were applied to confirm specificity against clades Ia and Ib. Additionally, non-mutated reference plasmids were utilized to verify lineage-specific detection of MPXV clade IIb A.1, B.1.

To account for inter-day and operational variability, experiments were performed in the three concentration points of 10^7^, 10^5^, and 10^3^ copies/μL by different operators over three days using distinct reagent lots. A cycle threshold (Ct) cut-off value of 40.0 was defined for positive detection.

### Clinical evaluation of the mrt-qPCR assays

Clinical evaluation of the mrt-qPCR assays was analysed using 109 positive specimens, 1 clade IIb B.1 virus strain (Viral nucleic acid from the Emergency Technology Center of the CDC Virus Institute in China) and 15 negative specimens. These samples were analysed by commercial qPCR and NGS, then statistically analysed using R 4.3.3 software.

## Results

### Evolutionary characteristics of MPXV and probes design for A.1, B.1 and C.1 lineages

To elucidate the design strategies for lineage-specific probes, we conducted an analysis of the evolutionary characteristics of MPXV clade IIb and summarized the SNP sites that define its various lineages (**Figure 2**). MPXV clade IIb has been classified into dozens of distinct lineages, including A (primarily A.1, A.2, and A.3). The A.1 lineage further evolved into B.1 and C.1, which recently diversified into lineages D, E, and F (**Figure 2A**). Each of these lineages possesses unique SNP sites. Based on these sites, we designed probes incorporating LNA modifications to enable rapid detection of several major lineages (A.1, B.1, and C.1). Specifically, we developed probes targeting the unique sites of A.1 (C83326T, reference genome: MT903342), B.1 (G77383A, reference genome: MPXV_USA_2022_MA001), and C.1 (G64426T, reference genome: MPXV/human/Japan/Tokyo/2022/TKY220165) for lineage-specific detection (**Figure 2B**). The sequences and modification sites are provided in **Supplementary Table S1**.

### Sensitivity of the mrt-qPCR

**Figure 3:**
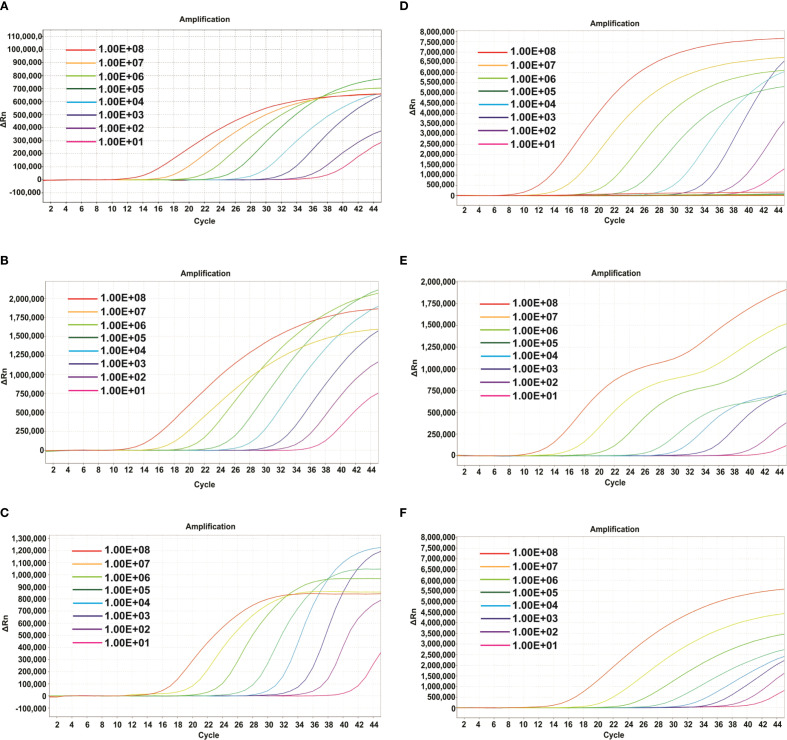
Amplification curves of different plasmids of MPXV(three replicates per dilution). In Panel 1. **(A-C)** The amplification curve of the different dilutions of plasmids of MPXV clade Ia **(A)**, clade Ib **(B)** and clade II **(C)**. In Panel 2. **(D-F)** The amplification curve of the different dilutions of plasmids of lineage A,1 **(D)**, lineage B.1 **(E)** and lineage C.1 **(F)**.

To evaluate the detection sensitivity of the mrt-qPCR, the probes of clade Ia, clade Ib, and clade II were labeled with Fam, Hex and Cy-5, respectively. The plasmids were then detected with a 10-fold gradient dilution (1×10^8^-1×10^1^ copie/μL). The results showed that the sensitivities of clade Ia, clade Ib, and clade II were 33, 34 and 34 copies/reaction (**Figures 3A-C**). In panel 2, the sensitivity of the assay was similar to that of panel 1, which was also 69, 34 and 62 copies/reaction (**Figures 3D-F**; **Supplementary Table S3**).

**Figure 4:**
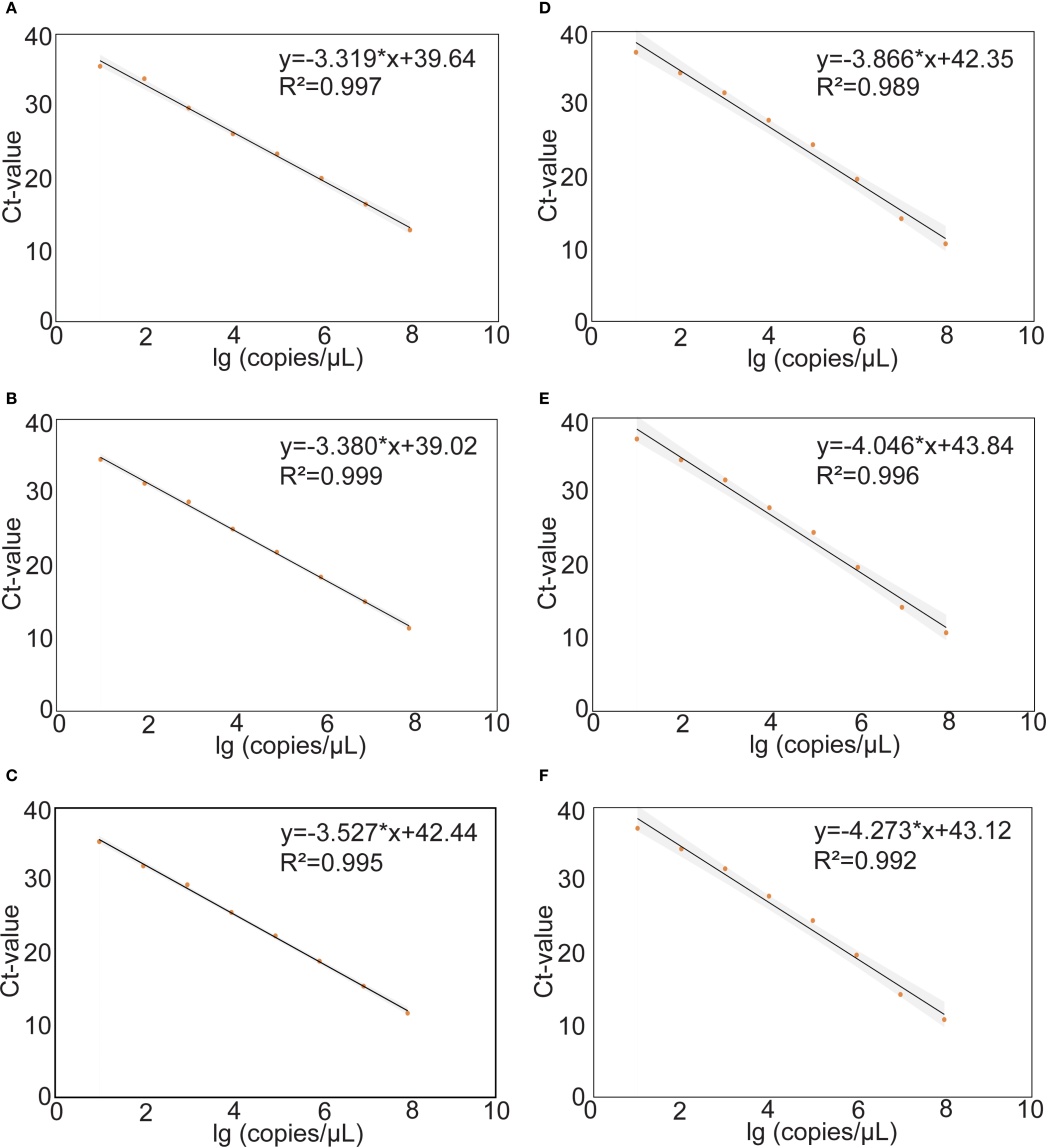
Standard curves of the different plasmids of MPXV. The standard curves were plotted against the log (copies/μL) against the Ct value from tenfold. The regression equation and R^2^ are shown on each curve. **(A-C)** Tenfold serial dilutions of purified plasmids DNA of the MPXV clade Ia **(A)**, clade Ib **(B)** and clade II **(C)**. **(D-F)** Tenfold serial dilutions of purified plasmids DNA of lineage A,1 **(D)**, lineage B.1 **(E)** and lineage C.1 **(F)**.

The construction of standard curves for mrt-qPCR assays is achieved by plotting the cycling threshold (Ct) value against the plasmid viral load (copies/μL). The standard curves were linear in the range of 10^8–^10^1^ copies for different MPXV subtypes, and the results showed that the standard curves performed a good linearity. In panel 1, the correlation coefficient (R^2^) of MPXV clade 1A, clade 1B, and clade II were 0.997, 0.999 and 0.995. In panel 2, the R^2^ of MPXV lineage A,1, lineage B.1, and lineage C.1 were 0.989, 0.996 and 0.992 (**Figure 4**).

### Specificity of the mrt-qPCR

To systematically evaluate the detection specificity of this method, a cross-reactivity validation experiment was designed in this study. The ectromelia virus (ECTV) and MPXV clade IIb B.1 virus and C.1 samples were used as detection targets to evaluate the detection specificity, and the results showed that there were not cross reaction with ECTV. MPXV clade IIb B.1 virus and C.1 samples could be detected only by the probe labeled Cy-5, there was no amplification signals by the probe labeled Fam and Hex (clade Ia and Ib probes). Plasmid controls not containing a specific mutation site were constructed for validation analyses. The experimental data showed that all plasmids that did not contain specific mutation sites did not generate amplification signals, confirming the ability of the probe to accurately detect its specific mutation sites. And there were also not cross-reaction with ECTV. When detecting MPXV B.1 virus, positive amplification signals were found in both lanes A and B, negative in lineage C.1 lane. The C.1 samples showed positive amplification in all lanes. It meets our judgment criteria and demonstrates excellent specificity.

### Reproducibility

**Figure 5:**
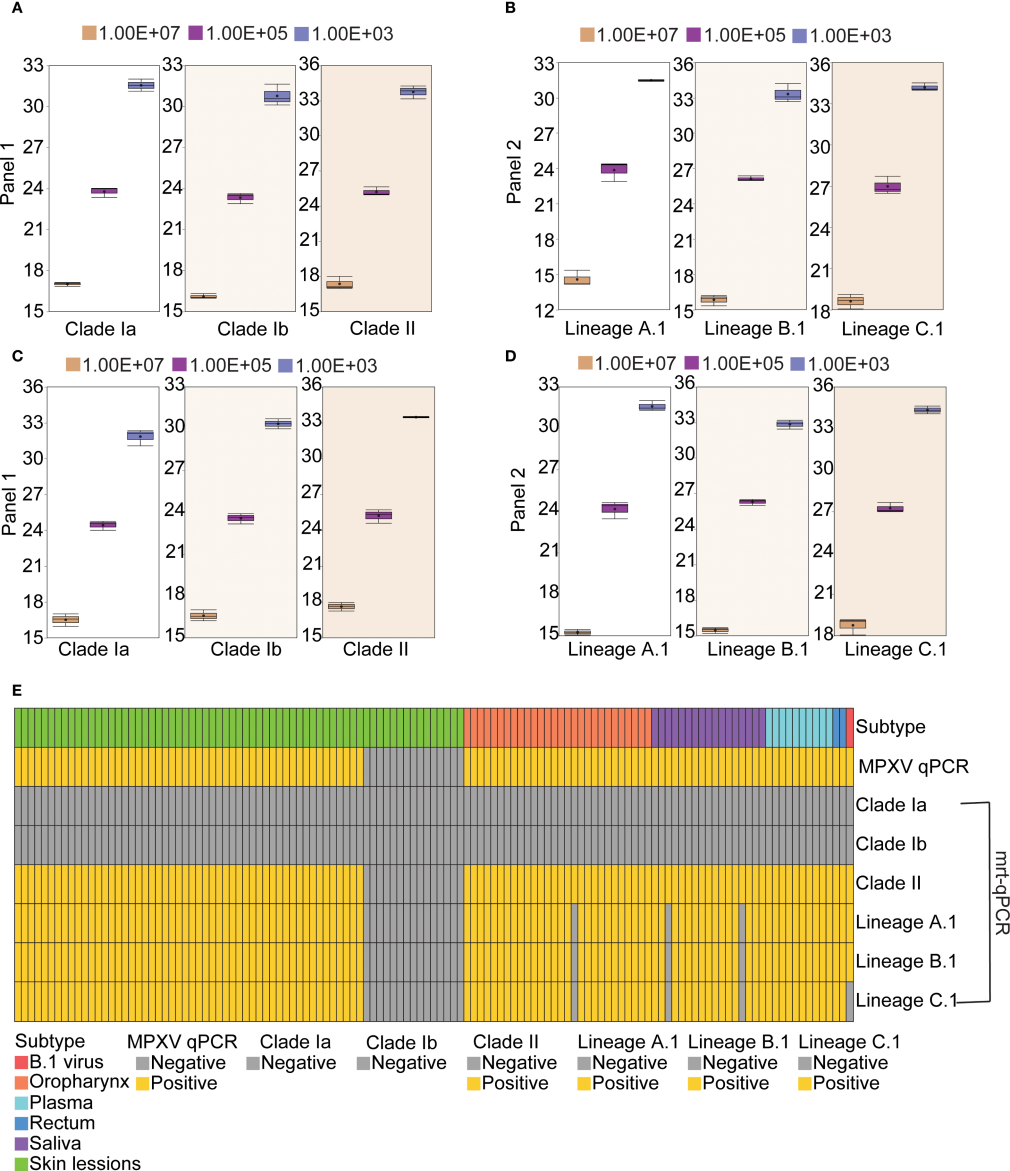
Reproducibility and clinical performance of the mrt-qPCR assay. **(A, C)** The intra-assay CV data **(A)** and inter-assay CV data **(C)** for panel 1. The box plots of Ct values for MPXV clade Ia, clade Ib and clade II at three concentration points (10^7^, 10^5^, and 10^3^ copies/μL) were shown. **(B, D)** The intra-assay CV data **(B)** and inter-assay CV data **(D)** panel 2. The box plots of Ct values for lineage A,1, lineage B.1 and lineage C.1 at three concentration points (10^7^, 10^5^, and 10^3^ copies/μL) were shown. **(E)** Distribution of results from 125 samples using different assays.

To assess the reproducibility of the mrt-qPCR assay, replicate validation of MPXV plasmids of different lineages with gradient dilutions (10^7^-10^3^ copies/μL) was performed on three independent experimental days. Three independent assays were performed for each of the three concentration points of 10^7^, 10^5^, and 10^3^ copies/μL. The results showed that the intra-assay coefficients of variation (CV) of the plasmids in panel 1 were 0.15%-2.66% (**Figure 5A**; **Supplementary Table S4**), and those in panel 2 were 0.64%-2.77% (**Figure 5B**; **Supplementary Table S5**), and the inter-assay CV of the plasmids in panel 1 were 0.83%-3.23% (**Figure 5C**; **Supplementary Table S6**), and those in panel 2 were 0.24%-4.66% (**Figure 5D**; **Supplementary Table S7**). The CV values of all assays were below the acceptable threshold of 5%. This shows that the established method has good stability and reproducibility.

### Clinical evaluation of the mrt-qPCR assays

A total of 109 qPCR positive specimens were collected, comprising swabs from 52 skin lessions, 28 oropharynx and 2 rectum, and 17 samples of saliva, 10 plasma samples (Yang et al., 2024). Additionally, a consistency analysis was performed on the evaluation methods for 15 qPCR negative specimens of skin lessions and 1 clade IIb B.1 virus strain. All positive specimens were confirmed to belong to MPXV C.1 through next-generation sequencing (NGS) (Zhang et al., 2024). The experimental results indicated that 109 qPCR positive specimens and 1 clade IIb B.1 virus strain were tested positive. No positive results were detected for clade Ia and Ib. Furthermore, a total of 106 specimens tested positive for MPXV C.1, specifically comprising 52 skin lessions, 27 oropharynx, 15 saliva, 10 plasma and 2 rectum samples. In Panel 1, the detection consistency for MPXV clade II was 100% (125/125) between the mrt-qPCR and qPCR. And in Panel 2, the detection consistency was 97.60% (122/125)) for C.1 lineage and 100% for B.1 lineage (**Figure 5E**; **Supplementary Table S8**).

## Discussion

Since May 2022, the MPXV clade II has continued to spread worldwide, with the emergence of variants such as clade IIb A.1, B.1, B.1.1, B.1.2, and C.1 (WHO Director-General declares mpox outbreak a public health emergency of international concern; Zhang et al., 2024). Notably, the more pathogenic clade Ib variant has been detected and transmitted in multiple countries and regions. While the current outbreak predominantly affectsMSM, the possibility of transmission among pregnant women and infants cannot be discounted (Chowdhury SR. et al., 2022; Dashraath et al., 2022; Hufstetler et al., 2024). Severe cases may lead to fatal outcomes. In comparison with previous MPXV epidemics, this outbreak demonstrates broader geographic spread and heightened transmissibility (WHO Director-General declares mpox outbreak a public health emergency of international concern). Consequently, accurate and rapid diagnostic tools are critical for timely outbreak containment and early clinical intervention.

Serological tests, such as neutralization and hemagglutination inhibition assays, have been developed for the detection of MPXV antibodies (Arita et al., 1972). However, the cross-reactivity of surface antigens among orthopoxviruses poses a significant challenge to overcome (Arita et al., 1972). Real-time PCR is a rapid, cost-effective, and highly sensitive method for the detection, differentiation, and monitoring of MPXV outbreaks (Altindis et al., 2022; Mills et al., 2023; Fan et al., 2024). Considerable efforts have been dedicated to the development of primers and probes targeting genes such as B6R, B7R, F3L, N3R, J7R, and O2L of MPXV, particularly to differentiate them from other orthopoxviruses (e.g., smallpox virus, cowpox virus, and varicella-zoster virus) (Ibrahim et al., 1997; Kulesh et al., 2004; Li et al., 2006; Maksyutov et al., 2016). However, the assays developed thus far are unable to further detect between the two clade of MPXV (clade I and clade II) and their respective subtypes. It is noteworthy that numerous existing qPCR assays targeting clade I demonstrate limited efficacy in detecting clade Ib (Schuele et al., 2024).

In this study, we successfully developed a two tube mrt-qPCR assay for the detection of various MPXV lineages. Notably, this assay is capable of detecting clade Ia, clade Ib, clade II, and their sub-lineages (MPXV A.1, B.1, C.1) with remarkable sensitivity (ranging from 33 to 69 copies/reaction) and specificity, and the results showed that the standard curves performed a good linearity. The sensitivity of this multiplex assay is nearly comparable to that of the singleplex qPCR for MPXV detection. Moreover, the multiplex method offers greater convenience for screening different MPXV lineages, while also saving time, labor, and costs (Huggett et al., 2022; Mills et al., 2023). Consequently, this method can efficiently and cost-effectively screen and differentiate between potential multinational or travel-associated transmissions of clade Ib and clade II. The CV of the plasmids in panel 1 were 0.83%-3.23%, and those in panel 2 were 0.24%-4.66%, this shows that the method has good stability and reproducibility. The high concordance rate between mrt-qPCR and conventional qPCR 100% for MPXV clade II in Panel 1 and 97.60% for C.1 lineages in Panel 2, which confirmed the reliability of the method. The assay’s versatility is underscored by its performance across various clinical samples, including swabs from skin lessions, oropharynx and rectum, and samples of saliva, plasma samples.

The LNA modified probes were used to enhance specificity and melting Tm values is particularly interesting because this method reduces cross-reactivity with closely related orthopoxviruses, such as cowpox virus and poxvirus (Chowdhury S. et al., 2022; Kamali et al., 2023). This strategy has been utilized in other virus detection systems to improve discrimination, as demonstrated by its application in tracking SARS-CoV-2 variants (Radvánszka et al., 2022; Shipulin et al., 2023). Additionally, the two tube multi-test system has been shown to simplify the workflow by isolating targets from specific evolutionary branches (Panel 1: Ia, Ib, II; Panel 2: MPXV A.1, B.1, C.1), thereby reducing the complexity of the assay while preserving diagnostic accuracy. Phylogenetic-specific detection of subevolutionary branches (e.g., B.1 was positive in both the A and B lane) is consistent with the evolutionary dynamics of MPXV, in which new lineages retain ancestral mutations. This feature not only enhances the monitoring of outbreaks, but also provides insights into viral transmission patterns. Nevertheless, continuous monitoring of the genomic diversity of MPXV as it evolves is imperative to ensure primer-probe validity. Genotyping of the MPXV relies on NGS and complex bioinformatics analysis, which is time-consuming, requires specialized expertise, and incurs high costs (Shipulin et al., 2023; Fan et al., 2024). This study addresses the detection of the novel clade Ib strain and clade IIb sublineages A.1, B.1, and C.1, filling a gap in lineage-specific diagnostic methods. In addition, the MPXV is primarily transmitted through MSM-a specific route of transmission. Although such a transmission pattern may result in relatively stable dominant strains of MPXV circulating in a given region (for instance, the dominant strain in Shenzhen from 2023 to 2024 was clade IIb C.1) (Angelo et al., 2023; Zhang et al., 2024), imported cases involving other strains still occur from time to time. Therefore, the rapid lineage identification method we have established, when combined with epidemiological analysis, serves as a crucial reserve measure for controlling the spread of the epidemic. Compared to NGS for typing of the MPXV virus (Zhang et al., 2024), the proposed approach offers rapid, accurate, and cost-effective advantages.

This study has same limitations. Due to the paucity of strains for clinical samples of MPXV Ia, Ib and A.1, the corresponding sample validation is lacking. and validation was conducted solely using plasmids. Additionally, although LNA-modified probes are more expensive than conventional probes, the extremely low consumption per reaction means the cost increase is minimal and does not limit its application in resource-limited settings. Furthermore, the detection of clade IIb A.1, B.1, and C.1 relies on a sequential detection pattern across three genetic loci, which is relatively more cumbersome compared to conventional qPCR assays. Addressing this limitation represents a key direction for our future research. Notably, this study also observed that in regions of high CG% content, the efficacy of LNA-modified probes was less pronounced even when multiple LNA modifications were incorporated. These findings underscore the importance of avoiding high CG%-content regions during LNA probe design.

## Conclusion

In conclusion, we have established a two-tube multiplex qPCR method for detecting different subtypes and lineages of the MPXV. This method addresses the issue of false-negative detection of MPXV clade Ib caused by gene fragment deletion, and has also enabled the development of a rapid detection approach for the predominantly circulating clade IIb (including lineages A.1, B.1, and C.1), thus filling the gap in the field. The mrt-qPCR assay provides a rapid, sensitive and scalable solution for the diagnosis and typing and lineage of MPXV. Moreover, the mrt-qPCR assay’s ability to detect MPXV in a variety of specimens, including those from different anatomical sites, underscores its versatility. This robustness and specificity of the assay make it a valuable tool for both clinical diagnostics and epidemiological surveillance.

## Data Availability

The raw data supporting the conclusions of this article will be made available by the authors, without undue reservation.

## Supplementary material

Supplementary Figure 1 Sequence alignments of target regions for the MPXV (clade Ia, clade Ib and clade II). **(A-C)** Sequences and positions of primers and probes for clade Ia **(A)**, clade Ib **(B)** and clade II **(C)**, respectively.
