# Supplementary figures and images for: Development of a two-tube multiplex real-time fluorescent PCR for the simultaneous differentiation of the mpox virus clades and the A.1, B.1 and C.1 lineages within clade IIb
Source: Front Cell Infect Microbiol. 2025 Oct 1;15:1611248. doi: 10.3389/fcimb.2025.1611248 (PMC12521436; doi:10.3389/fcimb.2025.1611248)

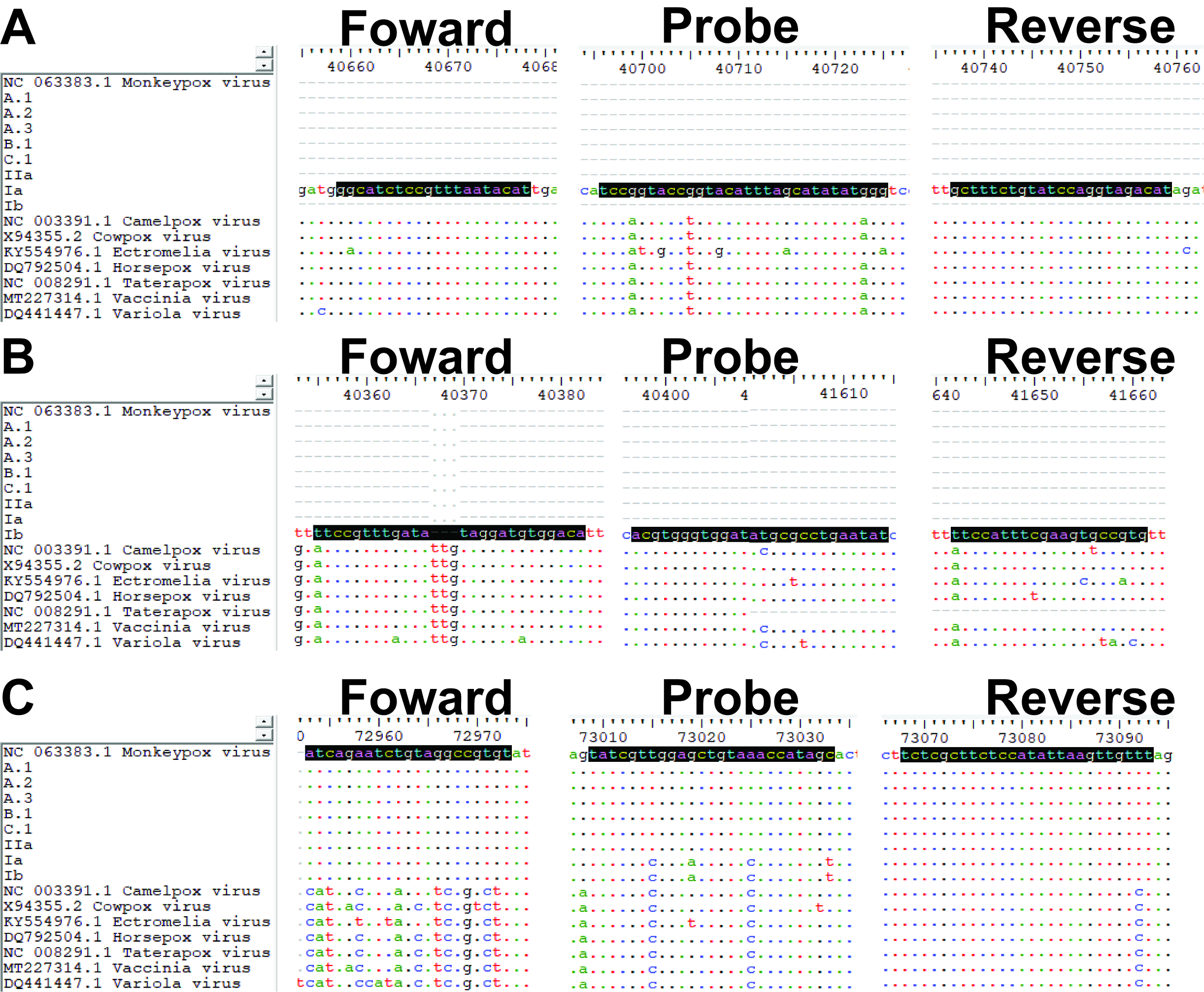

Supplement: Supplementary Figure 1 — Sequence alignments of target regions for the MPXV (clade Ia, clade Ib and clade II). (A-C) Sequences and positions of primers and probes for clade Ia (A), clade Ib (B) and clade II (C), respectively. [file Image1.tif]
