# Supplementary material for: Development of a two-tube multiplex real-time fluorescent PCR for the simultaneous differentiation of the mpox virus clades and the A.1, B.1 and C.1 lineages within clade IIb
Source: Front Cell Infect Microbiol. 2025 Oct 1;15:1611248. doi: 10.3389/fcimb.2025.1611248 (PMC12521436; doi:10.3389/fcimb.2025.1611248)
Supplement: Supplementary file 2 [file Table1.docx]

Table S1: Primers and probes of mrt-qPCR.

|  | **Name** | **Sequence** |
| --- | --- | --- |
| Panel 1 | clade Ⅰa F | TATGTCTACCTGGATACAGAAAGC |
|  | clade Ⅰa R | GGCATCTCCGTTTAATACATTGAT |
|  | clade Ⅰa P | Fam-CCCATATATGCTAAATGTACCGGTACCGGA-BHQ1 |
|  | clade Ⅰb F | TTCCGTTTGATATAGGATGTGGAC |
|  | clade Ⅰb R | CACGGCACTTCGAAATGGAA |
|  | clade Ⅰb P | HEX-ATATTCAGGCGCATATCCACCCACGT-BHQ1 |
|  | Clade Ⅱ F | ATCAGAATCTGTAGGCCGTGT |
|  | Clade Ⅱ R | AAACAACTTAATATGGAGAAGCGAGA |
|  | Clade Ⅱ P | CY5-CCCATATATGCTAAATGTACCGGTACCGGA-BHQ2 |
| Panel 2 | lineage A.1 F | AAGGATTAACGGGTTCTCAATATT |
|  | lineage A.1 R | ATGTTTCACAGACAATATCAGTAGA |
|  | lineage A.1 P | Fam-ACTTT/iXNA_T//iXNA_T//iXNA_G/ATGCTG-MGB |
|  | lineage B.1 F | AAGGATCATCACCTATCTTGTGT |
|  | lineage B.1 R | TCAAAGCCTTGTTGTCTCCGA |
|  | lineage B.1 P | HEX-TGTATAC/iXNA_T//iXNA_T//iXNA_A/GTCGATA-MGB |
|  | lineage C.1 F | CGGTATACTAGTAGGTAACTCAAAGAC |
|  | lineage C.1 R | TAGTAGCGATGAACTCGATCAA |
|  | lineage C.1 P | CY5-CGCG/iXNA_T//iXNA_T//iXNA_G/TTAAC-MGB |

Table S2: MPXV clade and lineages defining SNPs and reference

| Lineagesinformation | | | | Geneticcharacteristic | |
| --- | --- | --- | --- | --- | --- |
| Lineages | Parent lineage | Level | Years of find | Defining SNPs | Reference sequences |
| A | NA | Lineage | 2022 | 19367T,48527A | MPXV-M5312_HM12_Rivers, MPXV-M2940_FCT, MK783031 |
| A.1 | A | Major Lineage | 2022 | 83326T | MT903342, MT903343, MT903345 |
| A.1.1 | A.1 | Sublineage | 2022 | 34459A | MPXV_USA_2021_MD |
| A.2 | A | Lineage | 2022 | 34472T | MPXV/113/19 |
| A.2.1 | A.2 | Sublineage | 2022 | 25072T,140492C,179537T | MPXV_UK_2022_9000155, MPXV_USA_2022_FL001 |
| A.2.2 | A.2 | Sublineage | 2022 | 21991A,103019T,158424A | MPXV_USA_2022_PA0016, MPXV_UK_2022_9000166 |
| A.2.3 | A.2 | Sublineage | 2022 | 57284T,74226T | MPXV_USA_2021_TX, Monkeypox/PT0428/2022 |
| A.3 | A | Lineage | 2022 | 96841A,100971T | MPXV_Nig_2017_297957, MK783033 |
| B.1 | A.1.1 | Major Lineage | 2022 | 77383A | MPXV_USA_2022_MA001 |
| B.1.1 | B.1 | Sublineage | 2022 | 74360A | MPXV/Germany/2022/RKI025 |
| B.1.10 | B.1 | Sublineage | 2022 | 89906T,94798A | MPXV_UK_2022_9000147 |
| B.1.11 | B.1 | Sublineage | 2022 | 18133T,159277A | MpxV/human/USA/WA-UW-070499/2022 |
| B.1.12 | B.1 | Sublineage | 2022 | 182950T | MpxV/human/CAN/UN-NML-3448/2022, MPX_BhVKjm_9000233 |
| B.1.13 | B.1 | Sublineage | 2022 | 175093A | MPXV-USA-IL-IDPH-002, MpxV/human/USA/OH-UW-073812/2022 |
| B.1.14 | B.1 | Sublineage | 2022 | 36617A,159779T | MPXV/Germany/2022/RKI011 |
| B.1.15 | B.1 | Sublineage | 2022 | 149818A,151362A | MPXV/Germany/2022/RKI018 |
| B.1.16 | B.1 | Sublineage | 2022 | 9389T,161797A,185557C | DC_INS_MPX_0082_L, ANT-LDSP-ANT-MPX-24561-E |
| B.1.17 | B.1 | Sublineage | 2022 | 12169A,44960A | hMPX/Human/USA/CA-LACPHL-MA00085/2022 |
| B.1.18 | B.1 | Sublineage | 2022 | 141757T,43706T,124690C | MpxV/human/USA/WA-UW-083401/2022 |
| B.1.19 | B.1 | Sublineage | 2022 | 9963A,148268T | ID6821 |
| B.1.2 | B.1 | Sublineage | 2022 | 186165A | MPXV/Germany/2022/RKI124 |
| B.1.20 | B.1 | Sublineage | 2022 | 53326A,164385T,187620T | MPXV_USA_2022_TX0040 |
| B.1.21 | B.1 | Sublineage | 2022 | 22415A,132698T | MPX/Human/USA/CA-LACPHL-MA00153/2022 |
| B.1.22 | B.1 | Sublineage | 2022 | 34784T,101418T | MPXV/Germany/2022/ON/RKI903, MPXV/UZ_REGA_205/Belgium/2022 |
| B.1.23 | B.1 | Sublineage | 2022 | 27935A,64171G,73235T | WA-UW-288850, MPXV/PT0823/2024 |
| B.1.3 | B.1 | Sublineage | 2022 | 190660A | MPxV/Prague_001/2022/flye_medaka |
| B.1.4 | B.1 | Sublineage | 2022 | 34308A | MpxV/human/CAN/UN-NML-3214/2022, MpxV/human/CAN/UN-NML-2833/2022, MpxV/human/CAN/UN-NML-3532/2022 |
| B.1.5 | B.1 | Sublineage | 2022 | 70780T | MPXV/Germany/2022/RKI162 |
| B.1.6 | B.1 | Sublineage | 2022 | 111029A | LIM-INS-001 |
| B.1.7 | B.1 | Sublineage | 2022 | 25644T | MPXV_UK_2022_9000078 |
| B.1.8 | B.1 | Sublineage | 2022 | 5595A,191615T | MPXV/Germany/2022/RKI235 |
| B.1.9 | B.1 | Sublineage | 2022 | 181367A | Monkeypox/PT0043/2022 |
| C.1 | B.1.3 | Major Lineage | 2022 | 105923A,64426T,55133A | MPXV/human/Japan/Tokyo/2022/TKY220165 |
| C.1.1 | C.1 | Sublineage | 2022 | 21062T,149963T | MPV/Human/USA/CA-LACPHL-MA00563/2023, MPV/PT0706/2023 |
| D.1 | B.1.6 | Lineage | 2023 | 10945A,39515A,44627T,56276A | MPXV/Human/USA/CA-LACPHL-MA00408/2023 |
| E.1 | C.1.1 | Lineage | 2023 | 13563A,121394T,162280A | MPV/PT0732/2023 |
| E.2 | C.1.1 | Lineage | 2023 | 13563A,151847A,37738T | MPXV/PT0661/2023 |
| E.3 | C.1.1 | Lineage | 2023 | 142797T | MPXV/PT0627/2023 |
| F.1 | B.1.20 | Lineage | 2023 | 11668A,35132A,70414T,70666T,96938T | NY-NYCPHL-001231 |
| F.2 | B.1.20 | Lineage | 2023 | 161629A,22167T | hMpxV/USA/CA-CDPH-1M1000491/2023 |
| F.3 | B.1.20 | Lineage | 2023 | 174050T,180618T | hMpxV/USA/IL-RIPHL-MPXV-050-0081/2023 |
| F.4 | B.1.20 | Lineage | 2023 | 34277T,92382T,122551T,126919A,176643A | hMpxV/USA/IL-RIPHL-050-0153/2024 |
| F.5 | B.1.20 | Lineage | 2023 | 150831T,126169A | hMpxV/USA/CA-CDPH-1M1000492/2023, NY-NYCPHL-001278 |
| F.6 | B.1.20 | Lineage | 2023 | 146690A,159608T,168964A | MpxV/human/USA/WA-UW-097833/2023 |
| Defining SNP: A characteristic single nucleotide polymorphism (SNP) that distinguishes different lineages. | | | | | |
| Reference Sequence: The representative genomic sequence of a specific lineage. | | | | | |

Table S3. Assay data used for calculating the detection sensitivity.

|  | Copies/μL | 10^1^ | 10^2^ | 10^3^ | 10^4^ | 10^5^ | 10^6^ |
| --- | --- | --- | --- | --- | --- | --- | --- |
| Panel 1 | clade Ia | 16/20 | 20/20 | 20/20 | 20/20 | 20/20 | 20/20 |
|  | clade Ib | 13/20 | 20/20 | 20/20 | 20/20 | 20/20 | 20/20 |
|  | clade  II | 13/20 | 20/20 | 20/20 | 20/20 | 20/20 | 20/20 |
| Panel 2 | lineage A.1 | 6/20 | 20/20 | 20/20 | 20/20 | 20/20 | 20/20 |
|  | lineage B.1 | 13/20 | 20/20 | 20/20 | 20/20 | 20/20 | 20/20 |
|  | lineage C.1 | 7/20 | 20/20 | 20/20 | 20/20 | 20/20 | 20/20 |

Table S4. The intra-assay coefficient of variation (CV) of the mrt-qPCR in panel 1.

|  | 1.00E+07 | | | 1.00E+05 | | | 1.00E+03 | | |
| --- | --- | --- | --- | --- | --- | --- | --- | --- | --- |
|  | clade Ia | clade Ib | clade II | clade Ia | clade Ib | clade II | clade Ia | clade Ib | clade II |
| Ct values | 15.96 | 17.01 | 17.24 | 24.55 | 23.65 | 25.34 | 32.34 | 30.00 | 33.41 |
|  | 16.58 | 16.23 | 17.65 | 24.00 | 23.17 | 25.71 | 32.12 | 30.71 | 33.53 |
|  | 17.00 | 16.54 | 17.96 | 24.75 | 23.91 | 24.60 | 31.07 | 30.36 | 33.46 |
| Mean | 16.51 | 16.59 | 17.62 | 24.43 | 23.58 | 25.22 | 31.84 | 30.36 | 33.47 |
| S | 0.44 | 0.33 | 0.29 | 0.32 | 0.31 | 0.46 | 0.57 | 0.29 | 0.05 |
| CV% | 2.66 | 1.99 | 1.65 | 1.31 | 1.32 | 1.82 | 1.79 | 0.96 | 0.15 |

Table S5. The intra-assay coefficient of variation (CV) of the mrt-qPCR in panel 2.

|  | 1.00E+07 | | | 1.00E+05 | | | 1.00E+03 | | |
| --- | --- | --- | --- | --- | --- | --- | --- | --- | --- |
|  | lineage A.1 | lineage B.1 | lineage C.1 | lineage A.1 | lineage B.1 | lineage C.1 | lineage A.1 | lineage B.1 | lineage C.1 |
| Ct values | 15.06 | 15.52 | 18.06 | 24.4 | 26.00 | 27.64 | 32.01 | 33.19 | 34.06 |
|  | 15.47 | 15.21 | 19.06 | 24.64 | 26.51 | 26.98 | 31.31 | 32.91 | 34.62 |
|  | 15.23 | 15.68 | 19.17 | 23.47 | 26.37 | 27.06 | 31.47 | 32.45 | 34.32 |
| Mean | 15.25 | 15.47 | 18.76 | 24.17 | 26.29 | 27.23 | 31.6 | 32.85 | 34.33 |
| S | 0.18 | 0.2 | 0.52 | 0.49 | 0.22 | 0.29 | 0.3 | 0.31 | 0.22 |
| CV% | 1.18 | 1.29 | 2.77 | 2.03 | 0.84 | 1.07 | 0.95 | 0.94 | 0.64 |

Table S6. The inter-assay coefficient of variation (CV) of the mrt-qPCR in panel 1.

|  | 1.00E+07 | | | 1.00E+05 | | | 1.00E+03 | | |
| --- | --- | --- | --- | --- | --- | --- | --- | --- | --- |
|  | clade Ia | clade Ib | clade II | clade Ia | clade Ib | clade II | clade Ia | clade Ib | clade II |
| Ct values | 16.85 | 16.02 | 17.11 | 24.02 | 23.48 | 25.03 | 31.12 | 30.12 | 33.15 |
|  | 17.12 | 15.98 | 16.98 | 23.98 | 23.64 | 25.64 | 32.01 | 30.58 | 33.84 |
|  | 17.06 | 16.31 | 18.01 | 23.35 | 22.89 | 24.96 | 31.54 | 31.64 | 34.26 |
| Mean | 17.01 | 16.10 | 17.37 | 23.78 | 23.34 | 25.21 | 31.56 | 30.78 | 33.75 |
| S | 0.14 | 0.18 | 0.56 | 0.38 | 0.40 | 0.37 | 0.45 | 0.78 | 0.56 |
| CV% | 0.83% | 1.12% | 3.23% | 1.58% | 1.69% | 1.48% | 1.41% | 2.53% | 1.66% |

Table S7. The inter-assay coefficient of variation (CV) of the mrt-qPCR in panel 2.

|  | 1.00E+07 | | | 1.00E+05 | | | 1.00E+03 | | |
| --- | --- | --- | --- | --- | --- | --- | --- | --- | --- |
|  | lineage A.1 | lineage B.1 | lineage C.1 | lineage A.1 | lineage B.1 | lineage C.1 | lineage A.1 | lineage B.1 | lineage C.1 |
| Ct values | 14.17 | 15.33 | 18.06 | 24.39 | 26.04 | 27.71 | 31.55 | 33.07 | 34.07 |
|  | 15.36 | 16.21 | 19.12 | 24.31 | 26.37 | 26.48 | 31.4 | 34.21 | 34.52 |
|  | 14.20 | 15.98 | 18.67 | 22.9 | 25.98 | 26.76 | 31.5 | 32.68 | 33.98 |
| Mean | 14.58 | 15.84 | 18.62 | 23.87 | 26.13 | 26.98 | 31.48 | 33.32 | 34.19 |
| S | 0.68 | 0.46 | 0.53 | 0.84 | 0.21 | 0.64 | 0.08 | 0.80 | 0.29 |
| CV% | 4.66 | 2.88 | 2.86 | 3.51 | 0.80 | 2.39 | 0.24 | 2.39 | 0.85 |

Table S8. Agreement between the mrt-qPCR and qPCR.

|  | | mRT-qPCR | | qPCR | | Agreement |
| --- | --- | --- | --- | --- | --- | --- |
|  |  | Positive | Negative | Positive | Negative |  |
| Panel 1 | clade Ia | 0 | 125 | 0 | 125 | 100% |
|  | clade Ib | 0 | 125 | 0 | 125 | 100% |
|  | clade II | 110 | 15 | 110 | 15 | 100% |
| Panel 2 | Lineage A.1 | 0 | 125 | 0 | 125 | 100% |
|  | Lineage B.1 | 1 | 124 | 1 | 124 | 100% |
|  | Lineage C.1 | 107 | 18 | 110 | 15 | 97.60% |
