# Supplementary material for: Development of a two-tube multiplex real-time fluorescent PCR for the simultaneous differentiation of the mpox virus clades and the A.1, B.1 and C.1 lineages within clade IIb
Source: Front Cell Infect Microbiol. 2025 Oct 1;15:1611248. doi: 10.3389/fcimb.2025.1611248 (PMC12521436; doi:10.3389/fcimb.2025.1611248)
Supplement: Supplementary file 3 [file Table2.docx]

## Nucleic acid extraction

**TIANamp Genomic DNA Kit**

Protocol

Ensure that Buffer GD and Buffer PW have been prepared with appropriate volume of ethanol (96-100%) as indicated on the bottle and shake thoroughly.

1. Samples preparation:
2. For blood, please use 200 μl fresh, frozen or anticoagulant-adding blood. If less than 200 μl, please make up with Buffer GA to 200 μl.

**Note: If the blood volume is 0.3-1 ml, please refer to the following step: add 3 times volume of Red Cell Lysis Buffer (TIANGEN, Cat.no GRT122) to the sample (e.g., add 900 μl Red Cell Lysis Buffer to 300 μl blood), then close the cap and invert the tube. Stay the tube at room temperature (15-30°C) for 5 min, and centrifuge at 12,000 rpm (~13,400 × g) for 1 min, then discard the supernatant and add 200 μl Buffer GA to the precipitate and mix by pulse-vortex.**

1. Add 20 μl Proteinase K, mix thoroughly.

If the sample is blood and cultured cell, proceed to step 3 after adding Proteinase K.

1. Add 200 μl Buffer GB to the sample, mix thoroughly, and incubate at 70°C for 10 min to yield a homogeneous solution. Briefly centrifuge the 1.5 ml microcentrifuge tube to remove drops from the inside of the lid. Note: White precipitate may form when Buffer GB is added. They will not interfere with the procedure and will dissolve during the heat incubation at 70°C. If precipitates do not dissolve during heat incubation, it indicates that the cell is not completely lysed and may result in low yield of DNA and impurity of DNA.
2. Add 200 μl ethanol (96-100%) to the sample, and mix thoroughly for 15 sec. A white precipitate may form on addition of ethanol. Briefly centrifuge the 1.5 ml microcentrifuge tube to remove drops from the inside of the lid.
3. Pipet the mixture from step 4 into the Spin Column CB3 (in a 2 ml Collection Tube) and centrifuge at 12,000 rpm (~13,400 × g) for 30 sec. Discard flow-through and place the spin column into the Collection Tube.
4. Add 500 μl Buffer GD (Ensure ethanol (96-100%) has been added) to Spin Column CB3, and centrifuge at 12,000 rpm (~13,400 × g) for 30 sec, then discard the flow-through and place the spin column into the Collection Tube.
5. Add 600 μl Buffer PW (Ensure ethanol (96-100%) has been added) to Spin Column CB3, and centrifuge at 12,000 rpm (~13,400 × g) for 30 sec. Discard the flow-through and place the spin column into the Collection Tube.
6. Repeat Step 7.
7. Centrifuge CB3 with collection tube at 12,000 rpm (~13,400 × g) for 2 min to dry the membrane completely. Put CB3 column at room temperature 2-5 min to air dry the membrane completely. Note: The residual ethanol of Buffer PW may have some affection in downstream application.
8. Place the Spin Column CB3 in a new clean 1.5 ml microcentrifuge tube, and pipet 50-200 μl Buffer TE directly to the center of the membrane. Incubate at room temperature for 2-5 min, and then centrifuge for 2 min at 12,000 rpm (~13,400 × g). **Note: If the volume of elution buffer is less than 50 μl, it may affect recovery efficiency. The pH value of elution buffer will have a great effect on eluting, we suggest using Buffer TE or distilled water (pH 7.0 8.5) to elute gDNA. For long-term storage of DNA, eluting in Buffer TE and storing at -30~-15°C is recommended, since DNA stored in water is subject to acid hydrolysis.**
